# Supplementary material for: Relationship between GABA-Ergic System and the Expression of Mephedrone-Induced Reward in Rats—Behavioral, Chromatographic and In Vivo Imaging Study
Source: Int J Mol Sci. 2023 Jun 9;24(12):9958. doi: 10.3390/ijms24129958 (PMC10298564; doi:10.3390/ijms24129958)
Supplement: Supplementary file 1 [file ijms-24-09958-s001.zip › ijms-2417356-supplementary.pdf]

Supplementary materials for

Relationship between GABA-Ergic System and the Expression of Mephedrone-Induced Reward in Rats—Behavioral, Chromatographic and In Vivo Imaging Study

Olga Wronikowska-Denysiuk, Agnieszka Michalak, Anna Pankowska, Łukasz Kurach, Paulina Kozioł, Artur Łazorczyk, Katarzyna Kochalska, Katarzyna Targowska-Duda, Anna Boguszewska-Czubara, Barbara Budzyńska

- ## *Supplementary S1*

Table S1. The detailed results of power analysis for post-hoc two way ANOVA of baclofen-CPP experiment. Performed using IBM SPSS Statistics software.

Source	Type III Sum of Squares	df	Mean Square	F	Sig.	Partial Eta Squared	Noncent. Parameter	Observed Power[b]
Corrected Model	110883,681[a]	5	22176,736	3,155	0,018	0,293	15,776	0,828
Intercept	40425,343	1	40425,343	5,751	0,021	0,131	5,751	0,647
pretreatment	56219,173	1	56219,173	7,999	0,007	0,174	7,999	0,787
posttreatment	26545,395	2	13272,697	1,888	0,165	0,090	3,777	0,368
Pretreatment × posttreatment	23283,472	2	11641,736	1,656	0,204	0,080	3,313	0,327
Error	267090,846	38	7028,706					
Total	421533,810	44						
Corrected Total	377974,527	43						

[a.] R Squared = 0,293 (Adjusted R Squared = 0,200); [b.] Computed using alpha = 0,05.

- ## *Supplementary S2*

The alternative presentation of the CPP results, shown as the percentage of the time spent in the drug-paired compartment during post-conditioning test. The results have been calculated using the following formula:

CPP result *(percentage of the time spent in the drug-paired compartment during post-conditioning test)* = time spent in drug-paired context/(time spent in drug-paired context + time spent in VEH-paired context) × 100%

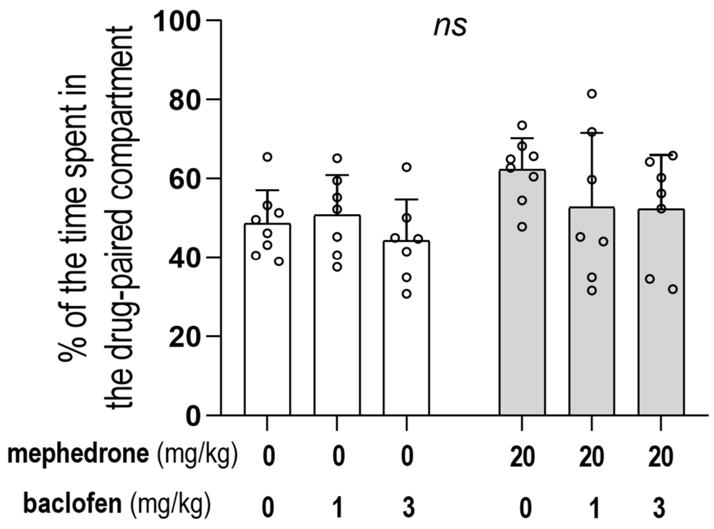

Figure S1 Percentage of the time spent in the drug-paired compartment during post-conditioning test for the experiment with baclofen. ns—no statistically significant difference.

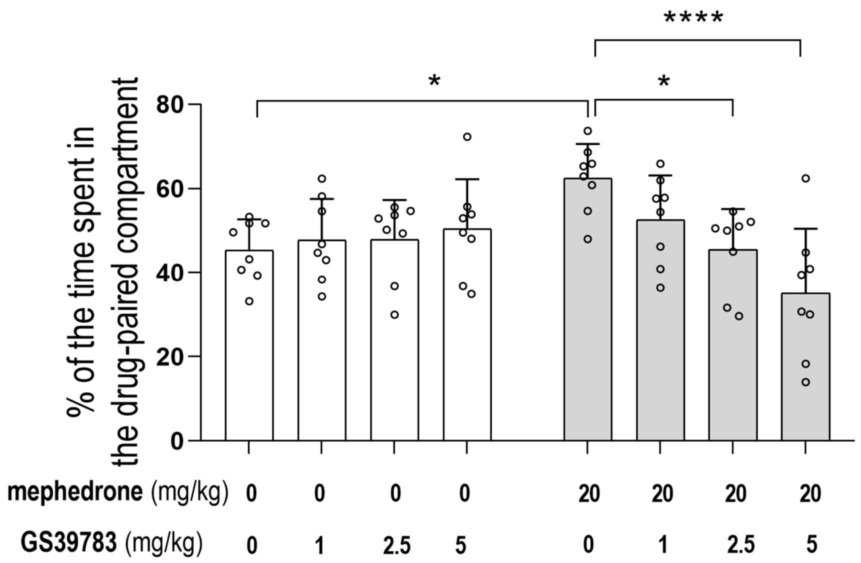

Figure S2 Percentage of the time spent in the drug-paired compartment during post-conditioning test for the experiment with GS39783; two-way ANOVA: [two-way ANOVA: GS39783 post-treatment: $F (3, 56) = 3.304$; $p = 0.0267$; mephedrone pre-treatment: $F (1, 56) = 0.1623$; $p = 0.6886$; interaction: GS39783 post-treatment × mephedrone pre-treatment: $F (3, 56) = 6.753$; $p = 0.0006$]. * $p < 0.05$; **** $p < 0.0001$.

- ## *Supplementary S3*

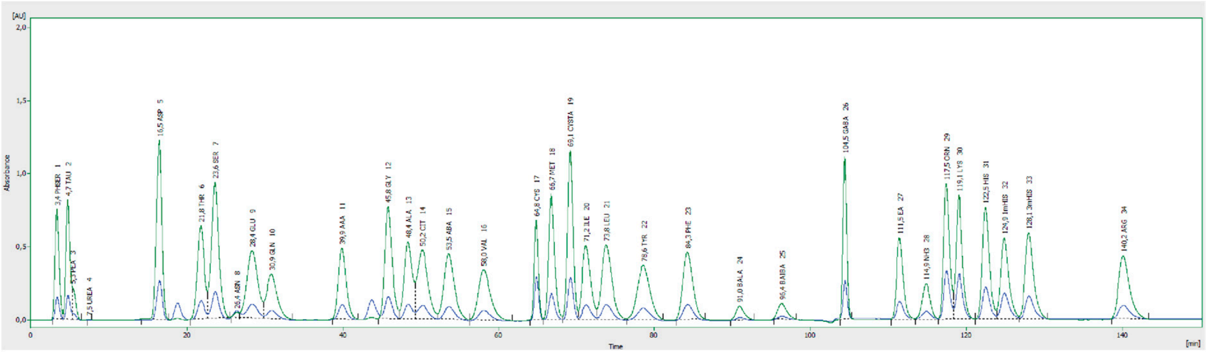

Figure S3 Exemplary chromatogram of standard solution at 250 nM concentration. Absorbance was registered at 440 and 570 nm

- ## *Supplementary S4*

The protocol applied for unbiased CPP protocol. In order to be qualified for an unbiased protocol, animals had to show a preference for each context in the maximum range of 40–60%.

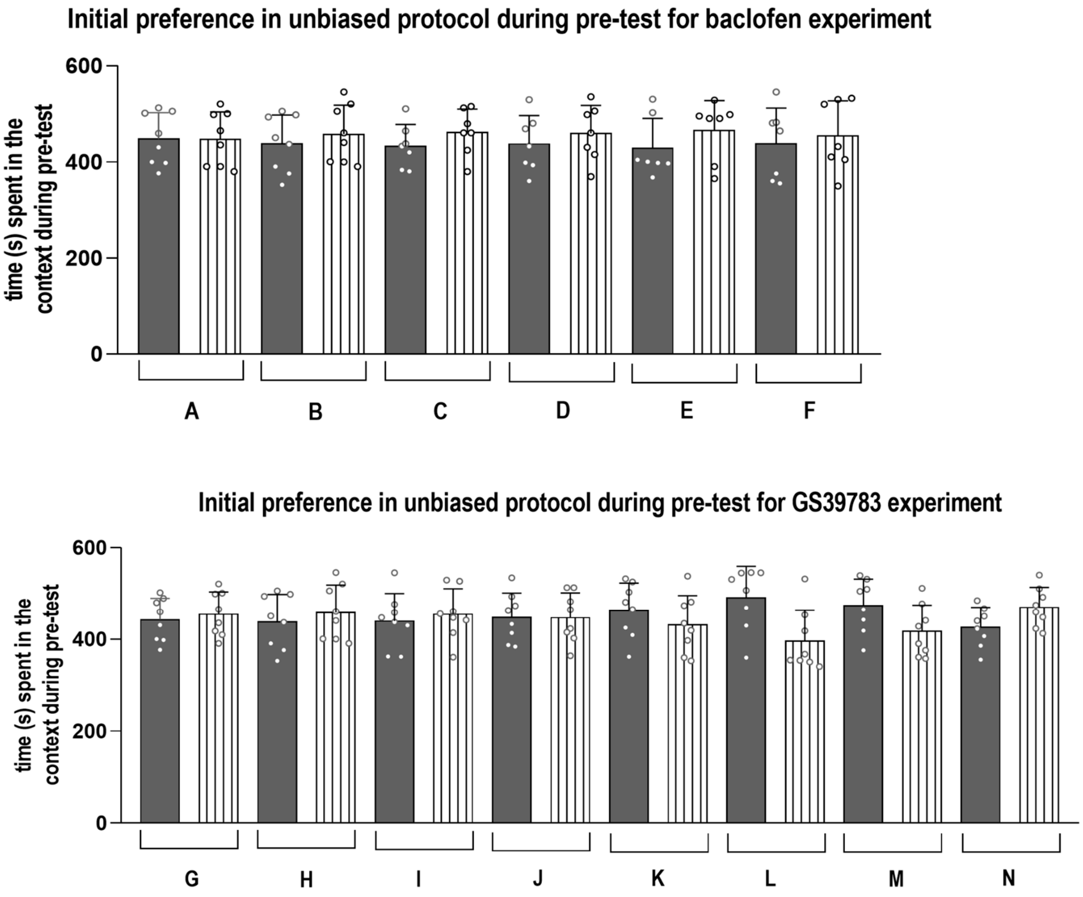

Figure S4. Initial preference for specific contexts measured during pre-test (*black* - black bars; *striped* - striped bars) of the final groups (see Table S1) assigned for the CPP experiment in unbiased protocol.

Table S2. Groups assigned for the CPP experiment along with the analysis of the time spent in specific context (black vs. stripes) during pre-test. *ns* - no statistical difference

Group	Conditioning Substance	Post-Conditioning Treatment	*t*-Test *(unpaired, two-tailed p value) black vs stripes within each group*	Statistics
**A**	saline	saline	$p = 0{,}9878$; $t = 0{,}01560$, df = 14	*ns*
**B**	mephedrone 20 mg/kg	saline	$p = 0{,}5149$; $t = 0{,}6682$, df = 14	*ns*
**C**	saline	baclofen 1.5 mg/kg	$p = 0{,}2645$; $t = 1{,}170$, df = 12	*ns*
**D**	saline	baclofen 1.5 mg/kg	$p = 0{,}4986$; $t = 0{,}6978$, df = 12	*ns*
**E**	mephedrone 20 mg/kg	baclofen 3 mg/kg	$p = 0{,}2849$; $t = 1{,}119$, df = 12	*ns*
**F**	mephedrone 20 mg/kg	baclofen 3 mg/kg	$p = 0{,}6866$; $t = 0{,}4134$, df = 12	*ns*
**G**	saline	saline	$p = 0{,}5972$; $t = 0{,}5407$, df = 14	*ns*
**H**	mephedrone 20 mg/kg	saline	$p = 0{,}5149$; $t = 0{,}6682$, df = 14	*ns*
**I**	saline	GS39783 1 mg/kg	$p = 0{,}6185$; $t = 0{,}5093$, df = 14	*ns*
**J**	saline	GS39783 2.5 mg/kg	$p = 0{,}9824$; $t = 0{,}02241$, df = 14	*ns*
**K**	saline	GS39783 5 mg/kg	$p = 0{,}3352$; $t = 0{,}9980$, df = 14	*ns*
**L**	mephedrone 20 mg/kg	GS39783 1 mg/kg	$p = 0{,}0532$; $t = 2{,}112$, df = 14	*ns*
**M**	mephedrone 20 mg/kg	GS39783 2.5 mg/kg	$p = 0{,}0721$; $t = 1{,}945$, df = 14	*ns*
**N**	mephedrone 20 mg/kg	GS39783 5 mg/kg	$p = 0{,}0624$; $t = 2{,}025$, df = 14	*ns*
